# Proteomic signatures corresponding to the SS18/SSX fusion gene in synovial sarcoma

**DOI:** 10.18632/oncotarget.26493

**Published:** 2018-12-25

**Authors:** Midori Ishii, Yoshiyuki Suehara, Kei Sano, Shinji Kohsaka, Takuo Hayashi, Saiko Kazuno, Keisuke Akaike, Kenta Mukaihara, Youngji Kim, Taketo Okubo, Kazuya Takamochi, Fumiyuki Takahashi, Kazuo Kaneko, Tsuyoshi Saito

**Affiliations:** ^1^ Department of Orthopedic Surgery, Juntendo University School of Medicine, Tokyo, Japan; ^2^ Department of Medical Genomics, Graduate School of Medicine, The University of Tokyo, Tokyo, Japan; ^3^ Department of Human Pathology, Juntendo University School of Medicine, Tokyo, Japan; ^4^ Laboratory of Proteomics and Biomolecular Science, Research Support Center, Juntendo University School of Medicine, Tokyo, Japan; ^5^ Department of General Thoracic Surgery, Juntendo University School of Medicine, Tokyo, Japan; ^6^ Department of Respiratory Medicine, Juntendo University School of Medicine, Tokyo, Japan

**Keywords:** synovial sarcoma, proteomics, SS18/SSX

## Abstract

Synovial sarcoma (SS) is a malignant soft tissue lesion and most commonly arises in young adults. Chromosomal translocation t(X;18)(p11;q11) results in the formation of *SS18*/*SSX* by gene fusion of the SS18 gene on chromosome 18 to either *SSX1*, *SSX2*, or *SSX4* gene located on chromosome X, which is detected in more than 95% of SSs. Although multiple lines of evidence suggest that the *SS18/SSX* fusion is the oncogene in this tumor, the protein expression profiles associated with *SS18*/*SSX* have yet to be elucidated. In this study, we conducted proteomic studies using *SS18*/*SSX* knockdown in three SS cell lines to identify the regulated proteins associated with SS18/SSX in SS. Isobaric tags for relative and absolute quantitation (i-TRAQ) analyses identified approximate 1700–2,000 proteins regulated by the SS18/SSX fusion in each SS cell line. We also analyzed the three profiles to identify proteins that were similarly altered in all 3 cell lines and found 17 consistently upregulated and 18 consistently downregulated proteins, including TAGLN and ACTN4. In addition, network analyses identified several critical pathways including RUNX2 and SMARCA4. RUNX2 and SMARCA4 had the highest ranking in these identified pathways. In addition, we found that expression of TAGLN inhibited cell viability in SS cell lines. Our data suggest that the differentiation and cell growth of SS may be enhanced by the identified proteins induced by SS18/SSX. We believe that the findings obtained in the present functional analyses will help to improve our understanding of the relationship between SS18/SSX and malignant behavior in SS.

## INTRODUCTION

Synovial sarcoma (SS) accounts for 7%–10% of all soft tissue malignancies and most commonly arises in the extremities of young adults with a slight male predominance [1, 2]. SSs are high-grade sarcomas, since they lead to death in at least 25% of patients within the first 5 years after the diagnosis, despite advances in treatment [3]. Current therapeutic approaches are not particularly effective in the treatment of SSs, especially for patients with recurrence and metastasis [3, 4]. Therefore, a deeper knowledge of the genetic alterations and molecular mechanisms involved in SSs may allow for the development of novel therapeutic strategies. SS is characterized by recurrent chromosomal translocation of t(X;18)(p11.2;q11.2), which results in the fusion of the *SS18* gene on chromosome 18 to either *SSX1*, *SSX2* or *SSX4* gene located on chromosome X, giving the fusion protein product SS18/SSX [5–8]. In addition, an analogous translocation of *SSX4* is detected in less than 1% of cases [9, 8]. Multiple lines of evidence have suggested that the SS18/SSX fusion is an oncogene and the central genetic “driver” in SS; (i) its presence as a sole cytogenetic anomaly in up to one-third of cases [9, 10], (ii) the low frequency of additional mutations [9, 11], (iii) its preservation in metastatic and advanced lesions [9, 10], (iv) the death of synovial sarcoma cells upon SS18/SSX knockdown [9, 12], and (v) its ability to induce tumors in conditional mouse models with appropriate histology, gene expression, and immunophenotype with 100% penetrance [9, 13]. In particular, the expression of INI1/SMARCB1/BAF47 is low or absent, while that of NY-ESO-1, a cancer-testis antigen, is high in SSs, as previously mentioned [14–16].

Analyzing the SS18-SSX function has been difficult, given the lack of an appropriate model system as the origin cells of SS are unknown [17]. Furthermore, most studies on SS have relied on heterologous cell types of uncertain relevance to human disease, with findings suggesting that the expression of genes associated with SS18/SSX may differ by cell type [17–20]. Given that the cellular background is critical for determining the function of SS18/SSX fusion protein in human SS, gene expression studies have employed SS18-SSX silencing by siRNA using three SS cell lines and identified genes related to SS18/SSX and several candidates for novel molecular therapy [2, 17]. However, the protein expression profiles associated with SS18/SSX have yet to be elucidated.

In this study, to identify the regulated proteins associated with SS18/SSX and clarify the function of SS18/SSX, we conducted proteomic studies using SS18/SSX knockdown in SS cell lines. We also conducted network analyses based on the protein profiles and carried out functional analyses of an identified critical protein and pathway.

## RESULTS

### SS18/SSX knockdown in SS cell lines

**Figure 1:**
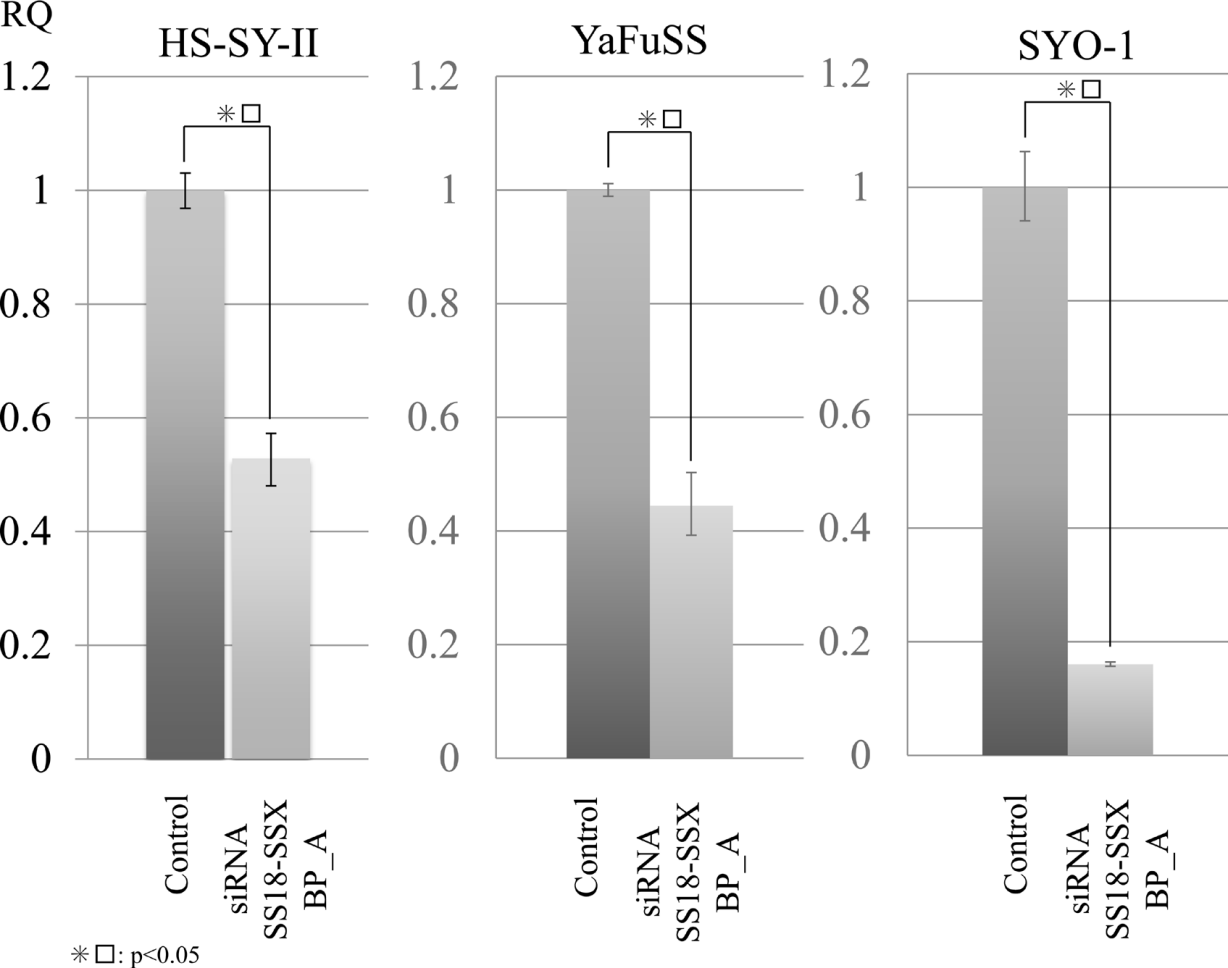
Proteomic analyses of SS18/SSX using SS18/SSX break point siRNA inhibited SS cell lines To identify protein profiles regulated by SS18/SSX, we performed i-TRAQ analyses using SS cells (HS-SYII, YaFuSS and SYO-1) transfected with siRNA against the SS18/SSX break point (SS18/SSX BP_A) or control. Before the proteomic analyses, we performed siRNA assays to generate these proteins. Quantitative PCR (qPCR) assays showed that siRNA SS18/SSX BP inhibited the mRNA expression of SS18/SSX in all three SS cell lines.

*SS18/SSX* knockdown was performed using three siRNA designed for the *SS18/SSX* break point (*SS18/SSX* BP_A, B and C). The SS18/SSX BP_A siRNA showed the strongest inhibition among these siRNAs in HS-SYII (Supplementary Figure 1). The SS18/SSX BP_A siRNA inhibited the SS18/SSX expression in all three SS cell lines, including the HS-SYII, YaFuSS, and SYO-1 lines (Figure 1 and Supplementary Figure 1). Regarding the cell proliferation, SS18/SSX knockdown with these siRNAs efficiently suppressed the cell proliferation of HS-SYII, YaFuSS, and SYO-1. We selected SS18/SSX BP_A siRNA to use in the subsequent experiments, as this siRNA showed the strongest effect.

### Identification of protein profiles associated with SS18/SSX expression in SS cell lines by i-TRAQ

To identify protein profiles associated with *SS18/SSX*, we performed i-TRAQ analyses using SS cells (HS-SYII, YaFuSS and SYO-1) transfected with siRNA against the *SS18/SSX* break point (*SS18/SSX* BP_A) or control. Proteins were extracted from both transfected SS cells. i-TRAQ analyses using each cell were carried out and identified 1976 (HS-SY II), 1976 (YaFuSS) and 1784 (SYO-1) or 1700-2000 proteins in each analysis. A statistical comparison led to the compilation of the protein profile, which differed significantly between the siRNA targets and control. In the SS cells with knockdown of *SS18/SSX* (Figure 1 and Supplementary Tables 1–3), analyses showed 33 downregulated proteins associated with *SS18/SSX* knockdown in HS-SYII, 36 in YaFuSS, and 34 in SYO-1 (*p* < 0.05). Furthermore, analyses showed 72 upregulated proteins associated with *SS18/SSX* knockdown in HS-SYII, 44 in YaFuSS, and 46 in SYO-1 (*p* < 0.05). We analyzed the 3 profiles to identify proteins that were similarly altered in all 3 cell lines and found 18 consistently upregulated and 17 consistently downregulated proteins, including TAGLN and ACTN4 (Table 1, Supplementary Figure 2 and Supplementary Figure 3).

**Table 1:** Proteins regulated by the SS18/SSX fusion gene

Accession no.	Symbol	Protein name	Fold difference	*P* value
Q01995	TAGL_HUMAN	Transgelin	2.32	0.00E+00
P09493-3	TPM1_HUMAN	Isoform 3 of Tropomyosin alpha-1 chain	2.02	3.20E-03
P06703	S10A6_HUMAN	Protein S100-A6	1.87	3.02E-02
P04792	HSPB1_HUMAN	Heat shock protein beta-1	1.65	1.88E-02
O43707	ACTN4_HUMAN	Alpha-actinin-4	1.49	2.80E-03
P51884	LUM_HUMAN	Lumican	1.41	1.67E-02
P67936	TPM4_HUMAN	Tropomyosin alpha-4 chain	1.34	2.00E-02
P49902-2	5NTC_HUMAN	Isoform 2 of Cytosolic purine 5'-nucleotidase	1.31	3.70E-02
P21333-2	FLNA_HUMAN	Isoform 2 of Filamin-A	1.29	1.33E-04
P08758	ANXA5_HUMAN	Annexin A5	1.29	2.50E-04
Q14315	FLNC_HUMAN	Filamin-C	1.25	1.70E-03
P35579	MYH9_HUMAN	Myosin-9	1.24	0.00E+00
Q99439	CNN2_HUMAN	Calponin-2	1.24	8.90E-03
P27797	CALR_HUMAN	Calreticulin	1.23	1.40E-03
P07237	PDIA1_HUMAN	Protein disulfide-isomerase	1.23	1.53E-03
P18206	VINC_HUMAN	Vinculin	1.22	2.10E-03
P29401	TKT_HUMAN	Transketolase	1.14	1.40E-02
P23284	PPIB_HUMAN	Peptidyl-prolyl cis-trans isomerase B	1.13	2.52E-02
P31948	STIP1_HUMAN	Stress-induced-phosphoprotein 1	0.90	9.75E-03
P50990	TCPQ_HUMAN	T-complex protein 1 subunit theta	0.89	1.61E-02
P49327	FAS_HUMAN	Fatty acid synthase	0.88	3.70E-03
P48643	TCPE_HUMAN	T-complex protein 1 subunit epsilon	0.88	7.43E-03
P17987	TCPA_HUMAN	T-complex protein 1 subunit alpha	0.87	3.17E-02
P78371	TCPB_HUMAN	T-complex protein 1 subunit beta	0.87	5.25E-03
P40227	TCPZ_HUMAN	T-complex protein 1 subunit zeta	0.87	2.10E-02
P09429	HMGB1_HUMAN	High mobility group protein B1	0.86	1.48E-02
P39023	RL3_HUMAN	60S ribosomal protein L3	0.84	6.80E-03
P08238	HS90B_HUMAN	Heat shock protein HSP 90-beta	0.83	1.73E-02
P53396	ACLY_HUMAN	ATP-citrate synthase	0.82	1.92E-02
P40429	RL13A_HUMAN	60S ribosomal protein L13a	0.82	1.35E-02
P50454	SERPH_HUMAN	Serpin H1	0.81	7.55E-03
P05141	ADT2_HUMAN	ADP/ATP translocase 2	0.79	2.15E-03
P26641	EF1G_HUMAN	Elongation factor 1-gamma	0.78	2.75E-03
P62750	RL23A_HUMAN	60S ribosomal protein L23a	0.76	2.55E-02
P62917	RL8_HUMAN	60S ribosomal protein L8	0.75	2.44E-02

### Network analyses according to the protein profiles of SS18/SSX

To further understand the biological processes and networks involved in tumor malignancy based on the proteins regulated by *SS18/SSX*, we performed network analyses using the Ingenuity Pathways Analysis (IPA) system (QIAGEN, Redwood City, CA, USA). We conducted analyses using the proteins associated with *SS18/SSX* knockdown in SS cell lines. In the network analyses based on the profiles of *SS18/SSX* knockdown in SS cells, we identified several pathways involving RUNX2 and SMARCA4 that played a critical functional role and acted as upstream regulators of these proteins known to be associated with the SYT/SSX expression in SS cells (Supplementary Tables 4 and Table 5).

### Confirmation of the mRNA expression in identified proteins and pathways

**Figure 2:**
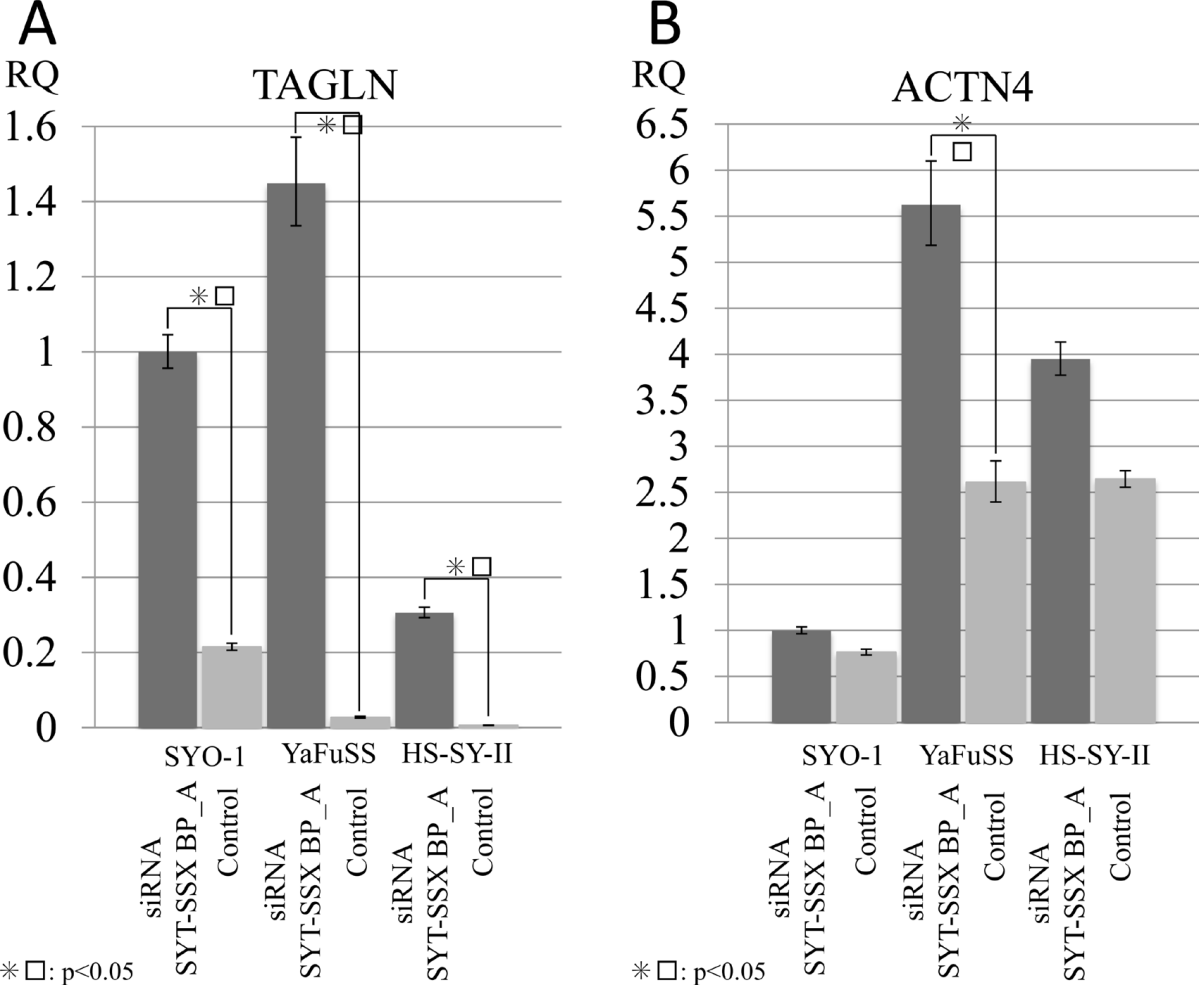
Silencing SS18/SSX activated the expression of TAGLN and ACTN4 To investigate the association of SS18/SSX with TAGLN and ACTN4, siRNA SS18/SSX was performed, and the mRNA expression was measured by q-PCR. The silencing of P SS18/SSX activated the expression of both TAGLN (**A**) and ACT4 (**B**) in SS cell lines (HS-SYII, YaFuSS, and SYO-1).

To confirm the mRNA expression corresponding to the identified proteins by proteomic analyses (i-TRAQ), we conducted qPCR of *TAGLN* and *ACTN4* using three SS cell lines (HS-SYII, YaFuSS, and SYO-1) that were transfected with *SS18/SSX* siRNA (siRNA *SS18/SSX* BP_A). Regarding *TAGLN*, the cell lines subjected to siRNA *SS18/SSX* knockdown showed significantly higher *TAGLN* expression than control samples (Figure 2A). Regarding *ACTN4*, the cell lines subjected to siRNA *SS18/SSX* knockdown had significantly higher mRNA levels of *ACTN4* than control samples (Figure 2B). These results suggest that our protein profiles for SS18/SSX were accurate.

With respect to IPA analyses, we identified several critical pathways including RUNX2 and CDKN2A. To confirm whether these findings matched the expression in SS, we investigated the mRNA status of both *RUNX2* and *CDKN2A* in SS18/SSX-regulated SS cells. The results showed that the expression of *RUNX2* and *CDKN2A* were consistently higher in all *SS18/SSX-*silenced SS cell lines than in control samples (Supplementary Figure 4). These results indicated that our protein profiles and pathway analyses had correctly identified proteins and transcript pathways that were regulated by SS18/SSX fusion.

### Effects of the TAGLN expression on the proliferation of SS cells

**Figure 3:**
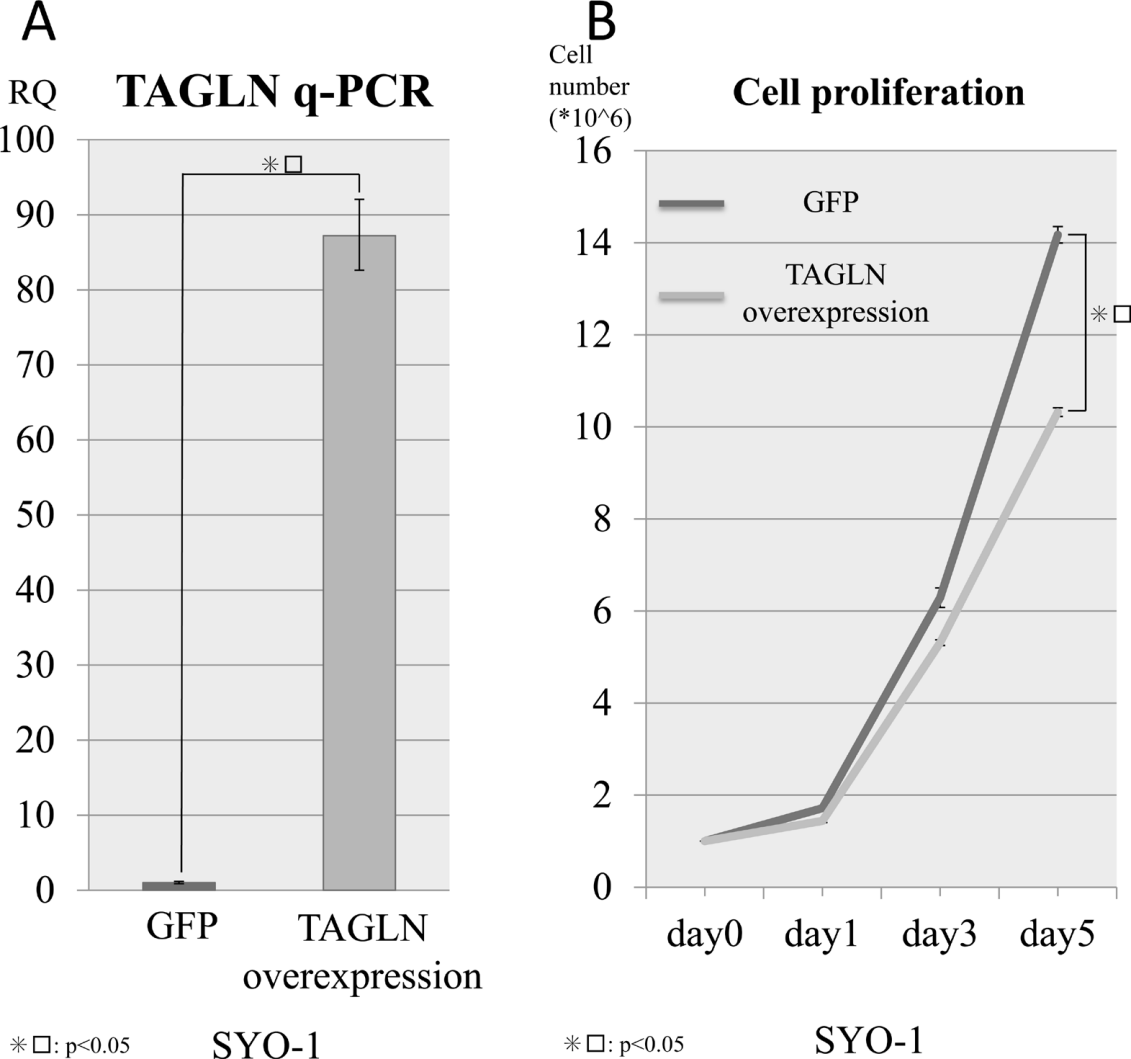
Cell viability with TAGLN overexpression TAGLN was transfected in SYO1 cells lines via retrovirus transduction to verify the associations between the TAGLN expression and cell viability. (**A**) q-PCR confirmed the overexpression of TAGLN in the SYO cell line and (**B**) the expression of TAGLN inhibited the cell viability in the SYO-1 cell line compared with control samples (GFP).

To investigate the association between TAGLN and the proliferation of SS cells, TAGLN forced expression was performed via TAGLN transfection in a SS cell line (SYO-1). q-PCR revealed that TAGLN-transfected cells had significantly higher *TAGLN* expression than control cells (GFP transfected cells) in the SYO-1 cell line (Figure 3A). In the cell proliferation assays, we also found that the cell proliferation was suppressed in SS cell lines by TAGLN expression (Figure 3B). These results revealed that silencing SS18/SSX suppressed SS cell growth in part via activation of *TAGLN* expression. Therefore, based on these findings, we concluded that our protein profiles contained crucial information regarding tumor malignancy and potential therapeutic targets in SS cells.

## DISCUSSION

Metabolic or chemical labeling was used for the quantitative proteomic analyses by mass spectrometry. Stable isotope labeling using amino acids in cell culture (SIALC) uses metabolic labeling and is applied only to cultured cells. In contrast, isobaric tag for relative and absolute quantitation (iTRAQ) uses chemical labeling and is able to label both biological samples and cultured cells with high efficiency. Regarding the quantification ability, both methods provide accurate results [21].

We conducted a proteomic study (iTRAQ) focusing on SS18/SSX fusion in SS and successfully identified several characteristic proteins as being associated with the SS18/SSX expression. To our knowledge, this study is the first report regarding the protein expression of SS18/SSX fusion in SS. Our protein profiles consistently identified 35 proteins regulated by the SS18/SSX expression across 3 SS cell lines. In these protein profiles obtained by the silencing of SS18/SSX fusion, 18 upregulated proteins that had the opposite expression to SS18/SSX and 17 downregulated proteins that had synchronous expression with SS18/SSX were identified.

Regarding to accuracy of our proteomic study, Heat shock protein (HSP) 90-beta and tropomyosin alpha 4 chain were also identified in both our SS18/SSX and SS surgical material studies [22]. HSP 90-beta was expressed in the group with a poor prognosis in the SS surgical material study and is listed among the proteins down-regulated by SS18/SSX. Tropomyosin alpha 4 chain was expressed in the group with a good prognosis in the SS surgical material study and is listed among the proteins up-regulated by SS18/SSX. These expression trends in these two proteins were coincident with both the surgical material and fusion gene profiles, suggesting that our protein profiles for SS18/SSX were accurate.

Among the 18 upregulated proteins, we detected several smooth muscle proteins, including transgelin (TAGLN), tropomyosin alpha-1 chain (TPM1), tropomyosin alpha-4 chain (TPM4), myosin-9 (MYH9), and calponin-2 (CNN2). TAGLN demonstrated the highest fold changes in our protein profile regulated by silencing SS18/SSX. A previous report found that TAGLN was specifically expressed in 154 of 184 leiomyosarcoma (LMS) (84%) and could be used as a novel diagnosis marker for tumor of smooth muscle differentiation, especially for LMS [23]. The high expressions of tropomyosin, myosin and calponin families have also been mentioned in proteomic expression studies as LMS specifically [24, 25]. Base on the previous studies and these identified proteins regarding SS18/SSX, our novel proteomic profile seems to indicate that SS18/SSX might have the ability to interrupt smooth muscle differentiation.

The cellular origin and differentiation of SS remain still unclear, but both mesenchymal and epithelial differentiation have been observed [26–28]. In gene expression studies, the high expression of neural lineage makers, including *NPTX2*, *NEFH*, and *NTNG2*, as well as chondrocyte lineage markers, including *COL2A1*, *COL9A3*, *SOX9*, and *TRPS1*, have been interpreted as consistent with differentiation from neural crest progenitors [9]. The high mRNA expression of *SOX2* also functions as a critical determinant of neural progenitors and embryonic stem cells in SS [9, 29, 30]. Several recent reports have suggested that SS is most likely derived from primitive mesenchymal cells that undergo differentiation [30–33]. In addition, regarding the primitive origin of SS, the expression of human SS18-SSX2 fusion protein within myoblasts in transgenic mouse model was shown to produce tumors similar to human SSs [31, 13, 34]. These present and previous findings suggest that SS18/SSX might suppress smooth muscle differentiation, while offering clues to the cellular origin and real differentiation of SS. In addition, in our confirmation studies using qPCR, silencing of SS18/SSX in SS activated the mRNA expression of TAGLN, which suppressed cell proliferation in SS cell lines. These findings suggested that our protein profiles also provide clues associated with tumor malignancy and identified several candidate therapeutic targets.

Gene expression studies regarding the regulators of SS18/SSX have identified several interesting genes [35, 12, 2, 17]. Takenaka *et al.* found that ACTN4, which our protein profiling also identified as upregulated by silencing SS18/SSX, reduced the malignant potential in SS [17]. ACTN4 performs crucial roles in signaling transduction, nuclear translocation, and gene expression regulation [36]. Honda *et al.* reported that ACTN4 contributed to cell motility and was highly expressed in breast cancer. In addition, ACTN4 is found in the membrane ruffles, and the inhibition of PI3 kinase (PI3K) promotes ACTN4 nuclear translocation in breast cancer and several cancer cell lines [37, 38]. These findings indicate that ACTN4 is closely associated with malignant potential in several cancers; however, interestingly, our proteomic study and previous gene expression studies have obtained the opposite findings in SS, namely showing that ACTN4 has the ability to suppress malignancy. Takenaka *et al.* also performed several functional studies focusing on adhesion including ACTN4 and ultimately concluded that SS18-SSX1 enhances adhesion to the extracellular matrix through the induction of expression of myosin light-chain kinase [17]. Our proteomic studies also identified myosin-9 as the myosin family. These findings might provide crucial information for clarifying the differences in the malignant behavior between epithelial cancers and SS. In addition, while other gene expression studies on silencing SS18/SSX in SS cells identified COM1 as a critical gene regulated by SS18/SSX and functioning as a cell proliferation regulator in SS, our protein profiling didn’t identify show that COM1 was such an abundant protein [35].

We also performed pathway analyses using the IPA software program and identified several crucial pathways, including runt-related transcription factor 2 (RUNX2) and SMARCA4. RUNX2 is also known as core-binding factor subunit alpha-1 (CBF-alpha-1) and is a key transcription factor associated with osteoblast differentiation in humans [39]. The radiologic characteristic of SS, found in 15%–20% of cases, is the presence of multiple small, spotty radiopacities caused by focal calcification and less frequently bone formation [1]. Of note, some reports have revealed that SSs with extensive calcification are associated with a good prognosis, and such calcifying SS cases have shown a 5-year survival rate of 82% [1, 40]. Furthermore, several histopathogenesis studies have explored the expression of RUNX2 in SS, and our finding of RUNX2 in the protein profile of SS was in accordance with the findings of those previous studies [1, 41, 42]. However, the histological effect of the RUNX2 expression on metaplastic calcification in SS is unclear, as SS18/SSX seems to suppress the RUNX2 expression in SS based on the data from these studies. Regarding SMARCA4, the SWI/SNF (BAF) complex includes SMARCA4, and the epigenetic modification of SS18/SSX alters the chromatin remodeling via epigenetic alterations through SWI/SNF(BAF)- and histone deacetylase (HDAC)-associated mechanisms [9, 43, 26, 44–47]. Recently, Kadoch described the high-affinity binding of SS18/SSX fusion to the core subunits of the SWI/SNF (BAF) complex and SS18 and SS18/SSX incorporate into the core subunits of SWI/SNF (BAF) complex, by dissociating BAF47 from the complex, thereby releasing SNF5, a tumor suppressor [43].

Other proteomic studies have recently been carried out for soft tissue sarcomas with specific fusion genes such as EWS/FLI1 (for Ewing sarcomas) and PAX3/FOXO1 (for alveolar rhabdomyosarcoma). These studies have identified numerous proteins; 90 proteins for EWS/FLI1 and 221 proteins for PAX3/FOXO1, despite this SS study have just identified 35 proteins for SS18/SSX [48, 49]. Several functional studies have found that SS18/SSX is not a transcriptional factor but co-activator [1, 9, 43, 26, 44–47]. In contrast, EWS/FLI1 and PAX3/FOXO1 were described as critical transcriptional factors in numerous previous studies [48, 49]. Therefore, based on these functional backgrounds, silencing of co-activator SS18/SSX picked up small amounts of regulated proteins compared to Ewing sarcoma and alveolar rhabdomyosarcoma having either EWS/FLI1 or PAX3/FOXO1 which act as transcriptional factors. With respect to differences in the expression of proteins that were regulated by these fusion genes, the characteristic genome status of SS18/SSX, a co-activator, might highlight slight differences in the expression of proteins regulated by SS18/SSSX compared to transcript factors (EWS/FLI1 and FOX3/FOXO1) [48, 49].

In the present study, we conducted proteomic analyses by silencing *SS18/SSX* in SS cells. Our data suggest that the differentiation and cell growth of SS may be enhanced by the identified proteins induced by SS18/SSX. We believe that our established protein profiles will help to improve the understanding of the relationship between SS18/SSX and the oncogenic behavior in SS, thereby leading to the development of novel therapeutic strategies.

## MATERIALS AND METHODS

### Cell lines

Three synovial sarcoma cell lines of HS-SY-II (Dr. Hiroshi Sonobe, Kochi University, Nankoku, Japan), YaFuSS (Dr. Junya Toguchida, Kyoto University, Kyoto, Japan), and SYO1 (Dr. Akira Kawai, Okayama University, Okayama, Japan) were kindly provided. All cell lines were grown in Dulbecco’s modified Eagle’s medium (DMEM) supplemented with 10% FCS and penicillin/streptomycin.

### RNA extraction, quantitative real-time PCR (q-PCR) and reverse transcription-polymerase chain reaction (RT-PCR)

RNA was isolated using an RNeasy Plus Mini kit (Qiagen, Hilden, Germany), and first-strand synthesis was performed using 5 μg of RNA and the SuperScript^®^ IV First-Strand Synthesis System (Thermo Fisher Scientific, Commonwealth, MA, USA). Quantitative real-time PCR (qPCR) was performed using inventoried Taqman assays from Applied Biosystems (Carlsbad, CA, USA) (20× Primer Probe mix) corresponding to SS18/SSX1 (Assay ID Hs03024820_ft), TAGLN (Assay ID Hs01038777_g1), ACTN4 (Assay ID Hs00245168_m1), RUNX2 (Assay ID Hs00231692_m1), CDKN2A (Assay ID Hs00923894_m1), ACTB (Assay ID Hs01060665_g1), and GAPDH (Assay ID Hs02758991_g1). All PCR reactions were performed with a TaqMan Fast Advanced Master Mix (Applied Biosystems) on an Applied Biosystems Step One Plus Real Time PCR System in accordance with the standard protocols. The amount of each target gene relative to the housekeeping gene ACTB and GAPDH was determined using the comparative threshold cycle (Ct) method. For each sample, the relative amount of each target gene was calibrated against a control transfected cell line. All assays were performed in triplicate. RT-PCR analyses were carried out to evaluate the expression of SS18/SSX using PCR SuperMix (Thermo Fisher Scientific). The human SS18/SSX primer sequences were as follows: 5′-CAGCAGAGGCCTTATGGATATGA-3′ and 5′-TTTGTGGGCCAGATGCTTC-3′. GAPDH was used as a loading control with the following primers: 5′-GAAGGTGAAGGTCGGAGTC3′ and 5′- GAAGATGGTGATGGGATTT-3′.

### SS18/SSX siRNA in SS cell lines

For the protein expression studies based on knockdown system, we used three cell lines (HS-SY-II, YaFuSS and SYO1). SS cell lines expressing SS18/SSX were treated with 50 nM of SS18/SSX break-point siRNA for A: Sense 5′-CCAGAUCAUGCCCAAGAAGdTdT-3′ Antisense 3′-dTdTGGUCUAGUACGGGUUCUUC-5′, B: Sense 5′-CCAGAUCAUGCCCAAGAAdTdT-3′, Antisense 3′-dTdTGGUCUAGUACGGGUUCUU-5′, C: Sense 5′-GACCAGAUCAUGCCCAAGAUU-3′, Antisense 3′-UUCUGGUCUAGUACGGGUUCU-5′, using Lipofectamine™ RNAiMAX reagent (Thermo Fisher Scientific). After 72 h, the total protein and RNA from each cell line was isolated, and the expression was validated by qPCR and reverse RT-PCR.

### Western blotting

The proteins were extracted from four SS cells, separated via SDS-PAGE and transferred to nitrocellulose membranes. The membranes were then incubated with either of the following antibodies: rabbit monoclonal antibodies against SS18/SSX (#70929, dilution 1:200; Cell Signaling, Danvers, MA, USA) or mouse monoclonal antibody against GAPDH (#sc-32233, dilution 1: 500; Santa Cruz, Dallas, TX, USA). After incubation, the membranes were washed 3 times with Tris-EDTA buffer and then reacted with horseradish peroxidase-conjugated secondary antibodies (dilution 1:1,000; GE Healthcare Biosciences, Boston MA).

### Isobaric tags for relative and absolute quantification (i-TRAQ) sample labeling, mass spectrometry analyses, and peptide identification

Analyses of proteins by i-TRAQ, a chemical label detected by mass spectrometry, were performed as described in our previous article (RR, SS). In brief, cell lysate samples was concentrated and buffer-exchanged using 3.5-kDa molecular weight cut-off spin concentrators (Tomy Seiko Co., Ltd., Tokyo, Japan) and then digested overnight with 10 µg L-1-(4-tosylamido)-2-phenylethyl tosylphenylalanyl chloromethyl ketone–treated trypsin. Each peptide solution was labeled with 1 of the 8 iTRAQ reagents (iTRAQ reporter ions of 113, 114, 115, 116, 117, 118, 119, and 121 mass/charge ratio) in accordance with the manufacturer’s protocol (AB SCIEX, Framingham, MA, USA). Labeled peptides were pooled and fractionated by strong cation exchange to four fractions. Each fraction was desalted with Sep-Pak C18 Plus Light Cartridge (Waters Corporation, Milford, MA, USA) and analyzed using nano liquid chromatography coupled with tandem mass spectrometry (LC-MS/MS); nano LC-MS was performed on a TripleTOF 5600 mass spectrometer for MS/MS operated with the Analyst TF 1.7 software program (AB SCIEX, Dublin, CA, USA) interfaced with the Eksigent nano LC system using a ChromXP C18-CL column (AB SCIEX). Protein identification and relative quantification was carried out using the ProteinPilot software program, version 5.0 (AB SCIEX). The functions of the various protein contents were determined by searching the UniProt database using the search algorithm within the ProteinPilot program (AB SCIEX). Protein ratios were normalized using the overall median ratio for all peptides in the sample for each separate ratio in every individual experiment. Two independent iTRAQ experiments were carried out to profile and quantitate the proteome, and the three technical replicates were used to determine the cut-off for significant fold-changes. A confidence cut-off of >95% was applied for protein identification, and a >1.2-fold change cut-off for all iTRAQ ratios was selected to classify proteins as up- or downregulated.

### Pathway analyses

The pathway analysis is shown in our previous article [50, 48]. In brief, the IPA software program (Qiagen) was further employed to determine the functional pathways of the identified genes. The IPA software program contains a database of biological interactions among genes and proteins and was used to calculate the probability of relationships among each of the canonical pathways, the upstream pathways, and the identified proteins. The IPA program scans the proteins that are entered by the user in order to identify networks using the Ingenuity Pathway Knowledge Base (IPKB) based on interactions between identified proteins and the known and hypothetical interacting genes stored in the program. The IPA program has been licensed for free use since 2013.

### Preparation of retrovirus and transduction of cell lines

For retrovirus production, the pcx4 (PMID: 14597713) and pGEM (Promega) vector systems were used. A plasmid encoding human TAGLN (Origene) was used to generate constructs, which were subcloned into pGEM, and the construct were subcloned into pCX4bleo. Retroviruses were obtained using αφ cells as packaging cells and infected into 3 SS lines for selection with 500 μg/ml zeocin (Thermo Fisher Scientific).

### Cell proliferation with TAGLN overexpression

To evaluate cell viabilities with the over-expression of TAGLN in SS, 1.0 × 10^6^ SS cells were plated into 100 mm dishes on day 0 with control (GFP). The cells were counted at 24 h, 72 h, and 120 h, and all proliferation experiments were performed in triplicate, with the results averaged.

### Statistical analyses

The relationships between the protein expression, cell proliferation, and other factors were analyzed using Fisher’s exact test or a *t-*test.

## SUPPLEMENTARY MATERIALS FIGURES AND TABLES










