# Supplementary material for: Proteomic signatures corresponding to the SS18/SSX fusion gene in synovial sarcoma
Source: Oncotarget. 2018 Dec 25;9(101):37509–19. doi: 10.18632/oncotarget.26493 (PMC6331019; doi:10.18632/oncotarget.26493)
Supplement: Supplementary file 1 [file oncotarget-09-37509-s001.pdf]

# Proteomic signatures corresponding to the SS18/SSX fusion gene in synovial sarcoma

## SUPPLEMENTARY MATERIALS

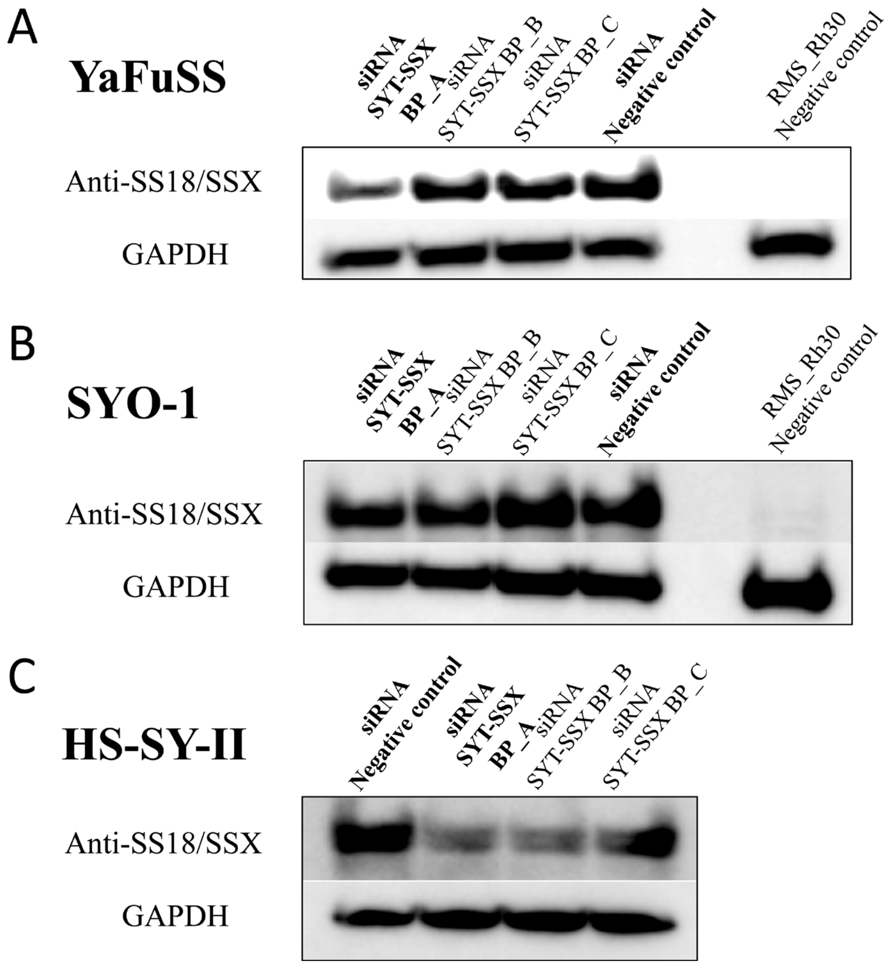


**Supplementary Figure 1: SS18/SSX break point siRNA inhibited the protein expression of SS18/SSX.** Western blotting was performed using proteins extracted from three SS cell lines (SS18/SSX1: HS-SYII and YaFuSS, SS18/SSX2: SYO-1 and negative control: RMS cell lines_Rh30) that were transfected with siRNAs targeting the SS18/SSX break point (using siRNA SS18/SSX BP_**A**–**C**). Quantitative PCR (qPCR) assays showed that siRNA SS18/SSX BP inhibited the mRNA expression of SS18/SSX in all three SS cell lines. SS18/SSX BP_A siRNA showed the greatest suppression of SS18/SSX protein expression.

| Accession no. | Symbol | Protein name | HS-SY-Ⅱ | YaFuSS | SYO-1 |
|---|---|---|---|---|---|
| Q01995 | TAGL_HUMAN | Transgelin | | | |
| P09493-3 | TPM1_HUMAN | Isoform 3 of Tropomyosin alpha-1 chain | | | |
| P06703 | S10A6_HUMAN | Protein S100-A6 | | | |
| P04792 | HSPB1_HUMAN | Heat shock protein beta-1 | | | |
| O43707 | ACTN4_HUMAN | Alpha-actinin-4 | | | |
| P51884 | LUM_HUMAN | Lumican | | | |
| P67936 | TPM4_HUMAN | Tropomyosin alpha-4 chain | | | |
| P49902-2 | 5NTC_HUMAN | Isoform 2 of Cytosolic purine 5'-nucleotidase | | | |
| P21333-2 | FLNA_HUMAN | Isoform 2 of Filamin-A | | | |
| P08758 | ANXA5_HUMAN | Annexin A5 | | | |
| Q14315 | FLNC_HUMAN | Filamin-C | | | |
| P35579 | MYH9_HUMAN | Myosin-9 | | | |
| Q99439 | CNN2_HUMAN | Calponin-2 | | | |
| P27797 | CALR_HUMAN | Calreticulin | | | |
| P07237 | PDIA1_HUMAN | Protein disulfide-isomerase | | | |
| P18206 | VINC_HUMAN | Vinculin | | | |
| P29401 | TKT_HUMAN | Transketolase | | | |
| P23284 | PPIB_HUMAN | Peptidyl-prolyl cis-trans isomerase B | | | |
| P31948 | STIP1_HUMAN | Stress-induced-phosphoprotein 1 | | | |
| P50990 | TCPQ_HUMAN | T-complex protein 1 subunit theta | | | |
| P49327 | FAS_HUMAN | Fatty acid synthase | | | |
| P48643 | TCPE_HUMAN | T-complex protein 1 subunit epsilon | | | |
| P17987 | TCPA_HUMAN | T-complex protein 1 subunit alpha | | | |
| P78371 | TCPB_HUMAN | T-complex protein 1 subunit beta | | | |
| P40227 | TCPZ_HUMAN | T-complex protein 1 subunit zeta | | | |
| P09429 | HMGB1_HUMAN | High mobility group protein B1 | | | |
| P39023 | RL3_HUMAN | 60S ribosomal protein L3 | | | |
| P08238 | HS90B_HUMAN | Heat shock protein HSP 90-beta | | | |
| P53396 | ACLY_HUMAN | ATP-citrate synthase | | | |
| P40429 | RL13A_HUMAN | 60S ribosomal protein L13a | | | |
| P50454 | SERPH_HUMAN | Serpin H1 | | | |
| P05141 | ADT2_HUMAN | ADP/ATP translocase 2 | | | |
| P26641 | EF1G_HUMAN | Elongation factor 1-gamma | | | |
| P62750 | RL23A_HUMAN | 60S ribosomal protein L23a | | | |
| P62917 | RL8_HUMAN | 60S ribosomal protein L8 | | | |

**Supplementary Figure 2: The heat map of the protein profiles regulated by SS18/SSX.** Proteins were up-regulated by siRNA SS18/SSX as labeled in red and down-regulated by siRNA SS18/SSX as labeled in blue.

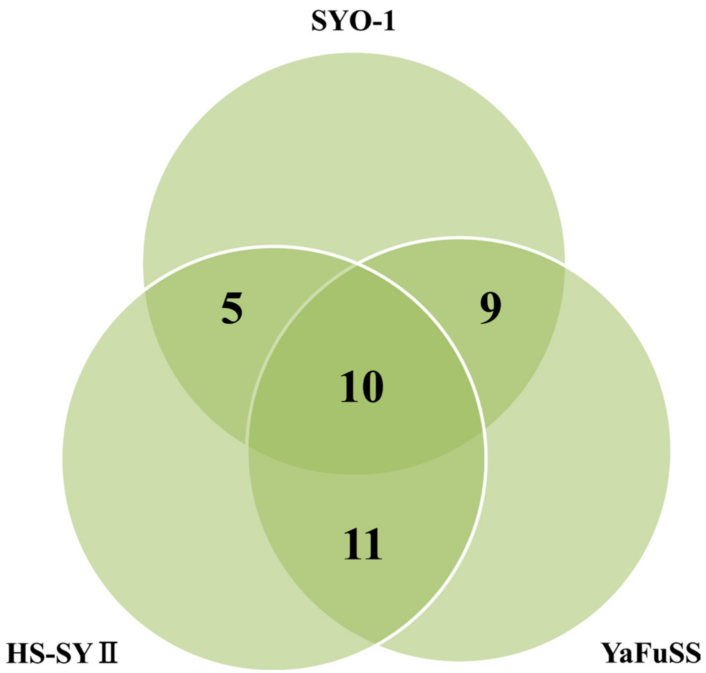


**Supplementary Figure 3: The Venn-diagram of proteins regulated by SS18/SSX in 3 SS cells.** Thirty-five proteins that were similarly altered in three SS cell lines were identified. Eleven of 35 proteins had similar alterations in all 3 SS cell lines.

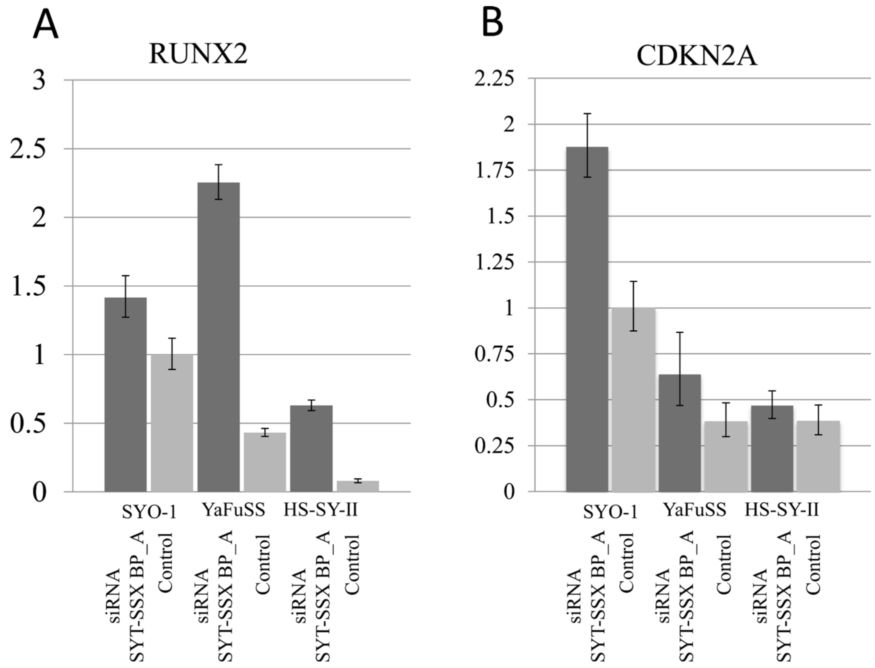


**Supplementary Figure 4: Silencing SS18/SSX activated the expression of RUNX2 and CDKN2A.** To investigate the association of SS18/SSX with RUNX2 and CDKN2A, siRNA SS18/SSX was performed, and the mRNA expression was measured by q-PCR. The silencing of SS18/SSX activated the expression of both RUNX2 (**A**) and CDKN2A (**B**) in SS cell lines (HS-SYII, YaFuSS, and SYO-1).

**Supplementary Table 1: Proteins regulated by the SS18/SSX fusion gene in HS-SY-II.** See Supplementary_Table_1

**Supplementary Table 2: Proteins regulated by the SS18/SSX fusion gene in YaFuSS.** See Supplementary_Table_2

**Supplementary Table 3: Protein regulated by the SS18/SSX fusion gene in SYO1.** See Supplementary_Table_3

**Supplementary Table 4: IPA analyses based on the protein profile of SS18-SSX**

| Upstream Regulator | Molecule Type | *p*-value of overlap | Target molecules in dataset |
|---|---|---|---|
| RUNX2 | transcription regulator | 3.84E-05 | LUM,TAGLN,TPM1 |
| SMARCA4 | transcription regulator | 1.67E-04 | ACTN4,LUM,TAGLN,TPM1 |
| SRF | transcription regulator | 4.11E-04 | MYH9,TAGLN,VCL |
| HSF1 | transcription regulator | 5.53E-04 | FASN,HSP90AB1,SERPINH1 |
| TP63 | transcription regulator | 1.40E-03 | FASN,TAGLN,TPM1 |
| THOC1 | transcription regulator | 1.73E-03 | CALR |
| NACC1 | transcription regulator | 3.46E-03 | HMGB1 |
| MYOCD | transcription regulator | 3.93E-03 | TAGLN,TPM1 |
| BARX2 | transcription regulator | 1.20E-02 | FLNA |
| SREBF2 | transcription regulator | 1.72E-02 | FASN |
| SMAD7 | transcription regulator | 1.89E-02 | TAGLN |
| ERG | transcription regulator | 2.15E-02 | FLNC,HMGB1 |
| SMARCA2 | transcription regulator | 2.73E-02 | TAGLN |
| IRF2 | transcription regulator | 2.90E-02 | MYH9 |
| IRF4 | transcription regulator | 3.41E-02 | PPIB |
| NFYA | transcription regulator | 3.41E-02 | FASN |
| MYC | transcription regulator | 3.52E-02 | FASN,HSPB1 |
| YAP1 | transcription regulator | 3.74E-02 | TAGLN |
| PPARGC1A | transcription regulator | 3.91E-02 | FASN |
| CDKN2A | transcription regulator | 4.07E-02 | TPM1 |
| RUNX1 | transcription regulator | 4.57E-02 | MYH9 |

**Supplementary Table 5: Network analyses of the expression profiles obtained using IPA.** See Supplementary_Table_5
